# Effect of Thiazolidinediones on the Erythropoeitic and Germinal Cells in the Male Wistar Rats

**DOI:** 10.4137/cmo.s678

**Published:** 2008-05-19

**Authors:** Syed Imam Rabbani, Kshama Devi, Salma Khanam

**Affiliations:** 1 Department of Pharmacology, Al-Ameen College of Pharmacy, Bangalore-560 027; 2 Department of Pharmacognosy, Al-Ameen College of Pharmacy, Bangalore-560 027

**Keywords:** rosiglitazone, pioglitazone, micronucleus test, sperm shape morphology

## Abstract

Hyperglycemia is the main determinant of long term diabetic complications mainly through induction of oxidative stress responsible for secondary defects including cancer, infertility etc. Thiazolidinediones (TZDs) are known to posses the antioxidant potential against the reactive oxygen species. The ability of clinically used TZDs like Rosiglitazone (RSG) and Pioglitazone (PIO) in diabetic complications is still need to be studied extensively in the literature. In this study, the role of RSG and PIO on the frequency of nuclear and germinal cell damage was studied using bone marrow micronucleus (MN) test, sperm shape abnormality and sperm count in normal animals. The drugs were tested in the three doses (1, 10 and 100 mg/kg) after acute (48 hrs and 72 hrs) and chronic (4 weeks) treatment. The results indicated that RSG has produced significant (p < 0.01) decrease in P/N (polychromatic and normochromatic erythrocytes) ratio at 10 and 100 mg/kg without affecting the frequency of micronucleated erythrocytes, sperm shape morphology and sperm count. PIO in the tested doses did not induce any change in P/N ratio and sperm count but the higher dose (100 mg/kg) showed suppression of MN in normochromatic erythrocytes and % sperm shape abnormality compared to the control group.

## Introduction

Diabetes mellitus is one of the common metabolic diseases, affecting about 3%–5% of the world’s population (Ramachandran et al. 2002). Increasing evidences in both experimental and clinical studies suggests that there is over production of reactive oxygen species (ROS) in the diabetes. Oxidative stress induces a variety of lesions in DNA, including oxidized bases, DNA strand breaks and formation of cross-links between DNA and proteins (Baynes, 1991). Mutations that occur due to DNA damage is known to cause cancer, heart ailments, aging and neurological diseases. The oxidative damage to the germinal cells is considered to be the leading cause for the various reproductive related complications resulting in infertility and other congenital and developmental defects (Singh et al. 2004). The mutagenic tests are increasing used as a reliable tool to measure the extent of DNA damage that occurs due to the environmental chemicals, drugs or diseases. Among the battery of tests available, the bone marrow micronucleus test in rodents is a well established method to assess the nuclear damage in the host system (Vijaylaxmi and Venu, 1999).

The pharmacotherapy of diabetes includes administration of insulin and/or oral hypoglycemics. Several classes of oral anti-diabetics are used; often combinations of these agents are administered to control the hyperglycemia. The chronic use of some of these agents has been reported to produce mutagenicity (Renner and Munzner, 1980).

Peroxisome proliferator activator receptor- gamma (PPAR-γ) ligands like Thiazolidinediones (TZDs) are the newer generation anti-diabetic agents primarily used to improve the peripheral insulin sensitivity (Berger and Moller, 2002). Among the TZDs, Troglitazone was the first to be approved by the USFDA, however it was later withdrawn from the market due to its association with severe hepatotoxicity (Kohlroser et al. 2000). Currently Pioglitazone (PIO) and Rosiglitazone (RSG) are the clinically used TZDs, both the compounds have the common thiazolidine-2-4-dione structure, differ in their side chain which may modify the pharmacological activity and side effects. Chemically PIO is 5-((4-(2-(5-ethyl-2-pyridinyl) ethoxy) phenyl) methyl) – 2, 4 – thiazolidinedione (C_19_ H_20_ N_2_ O_3_ S) whereas RSG is 5-((4-(2-(methyl – 2-pyridinylamino) ethoxy) phenyl) methyl) – 2,4 – thiazolidinedione (C_18_ H_19_ N_3_ O_3_ S) (Gillies and Dunn, 2000; Balfour and Plosker, 1999).

TZDs, apart from the anti-diabetic action, are reported to have several beneficial applications such as antioxidant, anticancer, anti-inflammatory etc (Saiki et al. 2007; Bao el al 2007; Galli et al. 2006; Chinetti et al. 2000). However, a recent study reveals that PIO administration for 2 weeks (10–40 mg/kg/day) has enhanced the DNA damage in rat hepatocytes and blood lymphocytes (Bedir et al. 2008). Similarly, in another study, RSG has shown increased nuclear damage in rat hepatocytes when it was tested daily (0.5–2.0 mg/kg) for two weeks (Bedir et al. 2006). Further, a product monograph also indicated that RSG treatment has produced a two-fold increase in mutations in the in vitro mouse lymphoma assay at toxic concentrations (150–200 μg/ml) (www.gsk/avandamet).

According to the OECD guidelines, the drugs that are used extensively and over a long duration of time need to be tested extensively for mutagenicity, carcinogenicity, teratogenicity and other types of complication on the host system (OECD guidelines for genotoxicity testing). Hence, the present research was planned to evaluate the role of clinically used TZDs on the mutagenic potential and to find their effect on the fertility related changes in male Wister rats.

## Materials and Methods

### Chemical

A gift sample of Rosiglitazone and Piogitazone were obtained from Biocon (India) Ltd, Bangalore. The stains and other reagents/chemicals used in this study were of analytical grade and procured from the regular suppliers. The acute and chronic treatment of the test drugs were done according to the guidelines for the mammalian micronucleus test (MacGregor et al. 1987).

### Animals

Eight week-old healthy, laboratory bred, male Wistar rats weighing 180 ± 10 gm were maintained under standard laboratory conditions such as temperature 22–25 ^o^C, 12 hour light/dark cycle and provided water and pellet food *ad libitum*. The experiments were conducted in CPCSEA (Committee for the purpose of control and supervision of experiments on animals, Chennai, India) approved animal house after obtaining the prior approval from the Institutional Animal Ethics Committee.

### Dosage, treatment and sampling

As per the guidelines to conduct the mammalian bone marrow micronucleus assay, at least three doses should be used to examine a dose-response relationship (MacGregor, 1987). In our study, we observed that daily administration of PIO and RSG to Wistar rats at a dose 100 mg/kg or above, past two weeks has shown symptoms of hypoglycemia like shivering, impaired response to auditory, visual and tactile response with occasional mortality (10%). Based on these observations, in the present study three doses viz., 1, 10 and 100 mg/kg were selected, 10 mg/kg being the reported therapeutic concentration (Sharabi et al. 2007; Gaikwad et al. 2007).

Forty animals were randomly selected and divided in to two groups namely; treatment and control. The treatment group received Rosiglitazone (1, 10, 100 mg/kg, p.o) and Pioglitazone (1, 10 and 100 mg/kg, p.o) suspended in 1% carboxy methyl cellulose (CMC). The samplings in the acute treatment were done after 48 hr and 72 hr. In the chronic group, the drugs were administered orally for 4 weeks and the sampling was done after 24 hr after the last dose. The control group received saline (1 ml/500 gm), simultaneously a solvent control was also performed that received 1% CMC (1 ml/500 gm).

### Bone marrow micronucleus (MN) test and scoring

The modified method of Schimid was followed to perform the bone marrow MN test (Vijaylaxmi and Venu, 1999). The animals after respective treatment were sacrificed by cervical dislocation under light anesthesia. Animals were cut open to excise femur and tibia. Bone marrow MN slides were prepared by using the modified method of Schmid. Marrow suspension from femur and tibia bones prepared in 5% bovine serum albumin (BSA), was centrifuged at 1000 rpm for 8 min and the pellet was resuspended in a required quantity of BSA.

**Plate 1:**
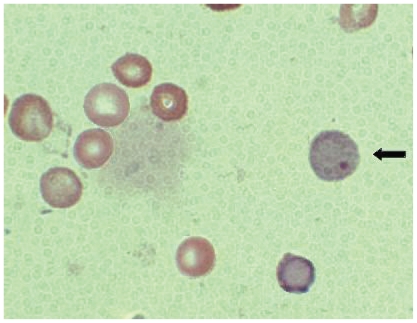
The arrow indicates micronucleated PCEs with normal NCEs.

A drop of this suspension was taken on a clean glass slide and smear was prepared on glass slide and air dried. The slides were fixed in absolute methanol, stained with May-Grunwald-Giemsa and MN were identified in two forms of RBCs (i.e. polychromatic erythrocytes as PCEs and normochromatic erythrocytes as NCEs) (Plate-1). About 2000 PCEs and corresponding NCEs were scanned for the presence of MN.

### Sperm morphology and sperm count assay

**Plate 2:**
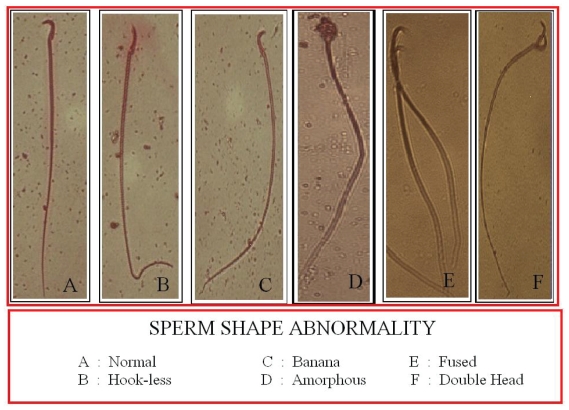
Represents different types of sperm shape abnormality.

The procedure described by Wyrobek and Bruce (1975) was followed to study the sperm shape abnormality. From the same sacrificed animals, cauda epididymis was immediately removed and minced in 10 ml phosphate buffer solution (PBS, pH 7.2) and the suspension was filtered through muslin cloth. To the filtrate, one drops of 1% eosin-Y was added and kept for 30 min for the staining of sperms. Smears were prepared on clean glass slides and air dried. One thousand sperms per animals were screened to find the different types of abnormality. Five types of abnormalities such as amorphous, hookless, banana shape, coiled/curved and double headed (Plate-2) were evaluated and finally represented as percentage total abnormality.

The sperm count test was performed according to Takizawa and Horii (2002). An aliquot of stained sperms solution was taken in white blood cell pipette up to the 0.5 mark and diluted further up to mark 11 with PBS. The mixture was shaken and charged in to the Neubauer’s chamber, the spermatozoal count was obtained by counting the number of sperm cells in the four WBC chambers using a neubauer’s slide.

### Statistical analysis

The statistical significance of the result was carried out using one-way ANOVA followed by Tukey multiple comparison test. P < 0.05 was considered to indicate the significance of the result.

## Results

### Bone marrow MN test

The ability of the drug treatment to cause the nuclear damage was tested using the bone marrow MN test. The administration of RSG (1, 10 and 100 mg/kg) in the acute treatment indicated that it did not cause any significant increase in the MN frequency nor altered the P/N ratio compared to control. However when RSG was tested for 4 weeks, there was significant (p < 0.01) variation in the P/N ratio. The % reduction in P/N ratio was found to be 13% and 22% at 10 and 100 mg/kg RSG treatment respectively. Additionally, the RSG treatment did not produce any difference in the number of micronucleated erythrocytes statistically. In the PIO group, the treatment in three doses did not produce any significant change in the P/N ratio. Conversely, PIO (100 mg/kg) exhibited a significant (p < 0.05) suppression of micronuclei formation in normochromatic erythrocytes when compared with normal group (Table 1).

### Sperm shape abnormality and sperm count

Three doses of RSG and PIO such as 1, 10 and 100 mg/kg were tested to assess their role on sperm shape abnormality and sperm count. Administration of RSG in these doses did not produce any significant variation in total percentage sperm shape abnormality and sperm count compared to control. However, the treatment with PIO showed a dose dependent decrease in the total number of sperm abnormality and the significant (p < 0.05) effect was observed at PIO 100 mg/kg dose. The treatment of PIO at the three doses tested did not affect the caudal sperm count (Table 2).

## Discussion

Bone marrow MN test in rodents is a established model to evaluate the cytogenetic damages caused by chemical and environmental mutagens. The induction of MN is studied in two forms of erythrocytes namely polychromatic and normochromatic erythrocytes to minimize the chances of counting artifacts as MN (Vijaylaxmi and Venu, 1999). Polychromatic erythrocytes are the immediate enucleated cells which still posses some nuclear material in the cytoplasm, enabling it to retain the nuclear stain. While normochromatic erythrocytes are the mature RBCs, devoid of the nuclear component, due to which they do not retain the nuclear stain during washing (Krishna and Hayashi, 2000). The ratio of P/N in normal condition is 1:1, decrease in the P/N ratio indicates the ability of the drugs to interfere in the cell cycle and inhibition in the proliferation of erythrocytes (Vijaylaxmi and Venu, 1999).

The present study indicated that RSG has shown significant reduction in P/N ratio and did not affect the MN frequency, sperm shape morphology and sperm count. On the other hand, PIO produced a significant decrease in the total percentage sperm abnormality without inducing any change in sperm count, micronucleated erythrocytes population and P/N ratio.

Earlier studies reveal that RSG induces cell growth arrest or death in normal and cancer cells. The possible mechanism suggested include that RSG inhibit the cancer cell growth in part through the activation of TSC2 (Tuberin) with the subsequent suppression of m TOR (molecular target of rapamycin) signaling (Sugimura et al. 1999; Hans et al. 2007). There have been reports that RSG might also cause disruptions in the cellular integrity by interfering in the transmembrane potential (Haskins et al. 2001). Involvement of similar mechanism in this study could have caused the suppression of proliferation of erythrocytes resulting in diminished P/N ratio. Further, a genotoxic study conducted on RSG indicated that exposure of rats to RSG has caused DNA damage largely in the liver cells. The authors suggest that generation of free radicals due to the cytochrome-mediated oxidation reactions could be the basis for the RSG induced nuclear damage (Bedir A et al. 2006). However, in the present work, RSG has not induced any nuclear damage as the frequency of MN erythrocytes did not increase significantly, indicating that RSG at the tested doses is non-mutagenic. In addition, the previous reports indicate that RSG in a series of in vivo and in vitro tests including the bacterial assays was found to negative for the gene mutation (www.gsk/avandamet).

Recently, a study conducted to evaluate the genotoxic potential of PIO indicated that the daily treatment of PIO to rats has increased the % tail DNA in hepatocytes and lymphocytes. Enhancement in the oxidative stress due to generation of free radicals from the mitochondrial and/or extra mitochondrial sources was considered as the possible reason for the PIO mediated nuclear damage (Bedir et al. 2008). However, several studies in the past have reported that TZDs posses potent anti-oxidant property. Both RSG and PIO are known to exhibit the antioxidant effect against free radicals by enhancing the endogenous antioxidant enzyme defence (Gumieniczek, 2005; Saiki et al. 2007; Bao et al. 2007). The ability of RSG and PIO to induce the DNA damage in the liver cells could be because; TZDs are primarily metabolized in liver by CYP P-450 enzymes, the oxidative stress that occurs due to the metabolism might have produced the localized damage to the hepatocytes DNA. However the PIO induced nuclear damage to the lymphocytes need to be further studied, especially in the context that TZDs are recognized to counteract the reactive oxygen species.

In this study, PIO did not produce any significant change in the P/N ratio. Moreover, administration of PIO (100 mg/kg) has reduced the number of micronucleated normochromatic erythrocytes compared to the control group. The ability of PIO to minimize the chromosomal damage can be attributed to its antioxidant property since inhibition in the free radicals action is known to suppress the nuclear damage (Saiki et al. 2007).

The cytonuclear damage to the reproductive cells results in infertility. In males, these damages cause both qualitative and quantitative defects in the spermatozoal cells. Changes in the sperm morphology not only decrease the fertility rate but also cause several developmental and birth defects (Singh et al. 2004). The oxidative stress due to the hyperglycemia is the principle factor in the diabetic patients responsible for the variation in sperm morphology and count. In addition, the chances of further increase in the damage due to the chronic administration of anti-diabetic agents can have negative impact on the patient physical and mental health (Kumar et al. 2002). Therefore, it becomes extremely imperative to test this category of drugs for their role on fertility. In this study, the tested doses of RSG and PIO after acute and chronic treatment did not show significant variation in the percentage sperm shape abnormality and sperm count. However, PIO at the highest tested dose (100 mg/kg) produced a significant reduction in the number of defective sperms. PIO in the earlier studies have been reported to enhance the level of antioxidant status (Saiki et al. 2007), this property of PIO, in this study too could have counteracted the actions of free radicals leading to decreased number of abnormal sperms.

Compounds possessing anti-proliferative and antidiabetic effects can be beneficial to the diabetic patients suffering from cancer (Galli et al. 2006). However, the data from the recent findings suggest that both RSG and PIO have the potential to cause genotoxic damage. These observations emphasis the need to test the safety of RSG and PIO by other battery of mutagenic tests, since many diabetic patients still use them to control the insulin resistance related complications world wide.

## Figures and Tables

**Table 1:** Effect of Rosiglitazone and Pioglitazone on bone marrow micronucleus frequency in normal Wistar rats after 48 hrs, 72 hrs and 4 weeks of treatment.

Treatment	Dose (mg/kg)	Micronucleus test after 48 hrs			Micronucleus test after 72 hrs			Micronucleus test After 4 weeks		
		% MN in PCEs	% MN in NCEs	P/N ratio	% MN in PCEs	% MN in NCEs	P/N ratio	% MN in PCEs	% MN in NCEs	P/N ratio
**Control** (Saline)	1ml/500 gm	0.34 ± 0.041	0.48 ± 0.032	1.01 ± 0.021	0.33 ± 0.017	0.42 ± 0.066	0.98 ± 0.025	0.41 ± 0.036	0.58 ± 0.087	1.10 ± 0.008
**Control** (Vehicle)	0.5 ml/kg	0.46 ± 0.037	0.50 ± 0.026	1.08 ± 0.081	0.41 ± 0.062	0.39 ± 0.042	1.11 ± 0.330	0.38 ± 0.017	0.47 ± 0.071	1.09 ± 0.052
**RSG**	1	0.40 ± 0.019	0.47 ± 0.046	1.02 ± 0.090	0.41 ± 0.082	0.46 ± 0.177	0.98 ± 0.091	0.45 ± 0.048	0.54 ± 0.084	1.02 ± 0.079
	10	0.39 ± 0.051	0.51 ± 0.049	1.02 ± 0.089	0.38 ± 0.047	0.51 ± 0.034	1.01 ± 0.069	0.42 ± 0.043	0.53 ± 0.090	0.97 ± 0.003^b^
	100	0.46 ± 0.102	0.48 ± 0.057	1.03 ± 0.031	0.42 ± 0.057	0.47 ± 0.048	1.035 ± 0.071	0.39 ± 0.032	0.55 ± 0.040	0.90 ± 0.029^c^
**PIO**	1	0.42 ± 0.041	0.45 ± 0.051	1.03 ± 0.160	0.43 ± 0.042	0.44 ± 0.030	1.12 ± 0.260	0.44 ± 0.011	0.50 ± 0.042	1.02 ± 0.120
	10	0.43 ± 0.061	0.42 ± 0.070	1.18 ± 0.183	0.41 ± 0.092	0.42 ± 0.034	1.04 ± 0.42	0.47 ± 0.071	0.47 ± 0.018	0.99 ± 0.062
	100	0.43 ± 0.085	0.50 ± 0.032	1.04 ± 0.050	0.41 ± 0.028	0.43 ± 0.019	1.03 ± 0.028	0.47 ± 0.064	0.44 ± 0.059^a^	1.04 ± 0.021

RSG-Rosiglitazone, PIO-Pioglitazone, MN-Micronuclei, PCEs-Polychromatic erythrocytes, NCEs-Normochromatic erythrocytes.

Values are expressed as Mean ± SD. N = 5.

Statistics: The group comparison was done by ANOVA followed by Tukey multiple comparison test

a **Note:** p < 0.05,

b p < 0.01,

c p < 0.001.

**Table 2:** Effect of Rosiglitazone and Pioglitazone on the sperm shape morphology and sperm count in normal Wistar rats after 48 hrs, 72 hrs and 4 weeks of treatment.

Treatment	Dose (mg/kg)	Sperm morphology test					
		After 48 hours		After 72 hours		After 4 weeks	
		Total% abnormality	Sperm count (10^6^)	Total abnormality	Sperm count (10^6^)	Total% abnormality	Sperm count (10^6^)
**Control** (Saline)	1 ml/500 gm	1.13 ± 0.060	33.18 ± 0.761	1.01 ± 0.410	33.02 ± 0.844	1.04 ± 0.158	31.07 ± 0.402
**Control** (Vehicle)	0.5 ml/kg	1.21 ± 0.190	32.34 ± 2.03	1.19 ± 0.081	31.29 ± 5.82	0.96 ± 0.011	34.33 ± 3.17
**RSG**	1	0.97 ± 0.124	33.25 ± 0.562	0.91 ± 0.045	33.07 ± 2.90	0.97 ± 0.090	31.5 ± 0.977
	10	0.97 ± 0.182	33.26 ± 2.91	0.96 ± 0.061	33.02 ± 0.80	0.89 ± 0.074	31.95 ± 2.08
	100	0.94 ± 0.291	30.16 ± 3.44	0.93 ± 0.052	32.98 ± 5.41	0.96 ± 0.102	31.77 ± 3.47
**PIO**	1	1.07 ± 0.075	33.27 ± 0.904	1.05 ± 0.023	33.23 ± 3.92	0.99 ± 0.074	31.15 ± 4.84
	10	1.05 ± 0.074	33.23 ± 1.38	1.05 ± 0.131	33.72 ± 3.08	0.88 ± 0.061	32.35 ± 1.26
	100	1.08 ± 0.103	32.5 ± 2.94	0.98 ± 0.390	32.41 ± 4.96	0.85 ± 0.084^a^	32.36 ± 2.32

RSG-Rosiglitazone, PIO-Pioglitazone.

Values are expressed as Mean ± SD. N = 5.

Statistics: The group comparison was done by ANOVA followed by Tukey multiple comparison test.

a p < 0.05.
